# Obstinate Vomiting Cured by Meat-Pancreas Injection

**Published:** 1879-02

**Authors:** 


Obstinate Vomiting Cured by Meat-Pancreas Injections.—In the case of a woman, forty-eight years of age, suffering from an abdominal aneurism, the vomiting was so persistent that the patient was unable to retain even a mouthful of water on her stomach. Dr. During, of Westhofen, under whose care she was, finally had recourse to Leube's nutritive clysters. Every day 1⅝ oz. of meat and ½ oz. of pancreas were chopped up very fine and mixed with warm water, until the compound had the consistency of a thin pap; half of this was injected into the rectum in the morning, and the other half in the evening, the clyster being retained each time for from eight to ten hours. The nutrition of the patient soon began to show signs of a slow improvement. After three weeks she was able to take a little milk by the mouth, but as the quantity thus taken did not exceed four tablespoonfuls per diem for several weeks, the progressive improvement could only be ascribed to the injections. After ten weeks the patient was so far improved that the clysters were discontinued. The gradual improvement in stomach digestion was accompanied by a progessive diminution in the size of the tumor.—*Med. Chir. Rundschau.—Med. Record.*

